# Classification of Amino Acids Using Hybrid Terahertz Spectrum and an Efficient Channel Attention Convolutional Neural Network

**DOI:** 10.3390/nano12122114

**Published:** 2022-06-20

**Authors:** Bo Wang, Xiaoling Qin, Kun Meng, Liguo Zhu, Zeren Li

**Affiliations:** 1 Quenda Terahertz Technologies, Ltd., 600 Jiushui E Rd., Qingdao 266102, China; wangbo@qdsrd.com; 2 School of Space Science and Physics, Shandong University, 180 Wenhua W Rd., Weihai 264209, China; 202017701@mail.sdu.edu.cn; 3 College of Engineering Physics, Shenzhen Technology University, 3002 Lantian Rd., Shenzhen 518060, China; zhuliguo@tsinghua.org.cn; 4 Institute of Fluid Physics, 64 Mianshan Rd., Mianyang 621900, China; lizeren@qdsrd.com

**Keywords:** terahertz spectroscopy, CNN, ECA, amino acid, sensor

## Abstract

Terahertz (THz) spectroscopy is the de facto method to study the vibration modes and rotational energy levels of molecules and is a widely used molecular sensor for non-destructive inspection. Here, based on the THz spectra of 20 amino acids, a method that extracts high-dimensional features from a hybrid spectrum combined with absorption rate and refractive index is proposed. A convolutional neural network (CNN) calibrated by efficient channel attention (ECA) is designed to learn from the high-dimensional features and make classifications. The proposed method achieves an accuracy of 99.9% and 99.2% on two testing datasets, which are 12.5% and 23% higher than the method solely classifying the absorption spectrum. The proposed method also realizes a processing speed of 3782.46 frames per second (fps), which is the highest among all the methods in comparison. Due to the compact size, high accuracy, and high speed, the proposed method is viable for future applications in THz chemical sensors.

## 1. Introduction

Terahertz time-domain spectroscopy (THz-TDS) has been applied to non-destructive testing on many substances due to its stronger penetration into dielectric materials compared with visible light and infrared [1,2,3]. In frequency domain, the effective band produced by desktop THz spectrometers ranges between 0.1 to 4 THz, and the upper limit of the frequency band can reach 6 THz using asynchronous optical sampling [4]. As the rotational energy levels and some of the vibrational modes of many biomolecules reside in the THz band, the pulse wave generated by the THz spectrometer is sensitive to the molecular structural changes and the binding between molecules, and thus creates a label-free approach to detect substances such as proteins, DNA, and explosives [5,6,7,8]. The molecular rotational resonance spectroscopy utilizes the unique THz spectral signatures of gas samples to analyze isotopic species [9]. However, due to the strong absorption of THz radiation by water and polar solvent, the restriction of dynamics range, and the significant scattering attributed to the ∼10 μm to mm wavelength of the THz pulses, many molecular features are difficult to extricate, which largely constrains the prospects of THz-TDS as a competent sensing technology [10,11,12]. Another challenge in applying the THz spectroscopy is that many molecular species generate non-distinguishable absorption spectra or even no characteristic features in the effective frequency band, which further restricts the identification based on THz spectroscopy [4,13].

Many methods have been proposed to extract the characteristic features from the THz spectrum. The principal component analysis (PCA) method is based on the idea that the majority of information of a multi-dimensional matrix can be represented by a few principal eigenvectors so that the minor features such as non-specific jitters are eliminated and matrices bearing similar features are easier to classify. Thereby, PCA can extract major features from the THz spectrum and has been used to identify drugs, soybean oil, and numerous chemicals from a large variety of substances [14,15,16]. In addition, partial least-squares (PLS) methods are used to select the absorption wavelength of the molecule and to find spectral intervals with the highest signal-to-noise ratios (SNR) [17,18], and hence concentrations of substances in mixtures are quantitatively estimated with high precision. Several variation of PLS including iPLS, biPLS, mwPLS, and siPLS are employed in the aforementioned studies. Additionally, the generic algorithm (GA) is a method to globally optimize parameters such as the absorption wavelength and to extract optical metrics such as refractive index and absorption rate from the data affected by noise, and is shown to produce better results than PLS and iterative algorithms [19,20,21]. However, these methods are based on numerous metrics of the data, and their performance decays largely for spectra with no apparent features.

Machine learning is the study of computer algorithms that can improve automatically through experience and by the use of data. As a novel branch of machine learning, neural networks can use numerous filters to extract the high-dimensional characteristics of the input data and study the interconnections between them, which makes it more adaptable to data lack of apparent features. In recent years, many researchers have used machine learning algorithms to classify the THz spectra. Hu et al. compared networks built with convolutional neural network (CNN) and recurrent neural network, and concluded that the identification based on CNN was more accurate and faster for a dataset containing 12 materials [22]. A network combining CNN and bidirectional gated recurrent network (BiGRU) was designed to trace the dynamic changes of the absorption spectrum in time domain, and overcame the difficulties of frequency–domain analysis such as band cutoff but took much longer time to process data due to the larger size of time signal [23]. However, previous research mainly focused on extracting features from 1D data such as absorption spectrum or time-domain spectrum, which did not fully integrate the amplitude and phase information of the pulsed THz wave, and hence restricts the accuracy of prediction. To date, no study associated with the classification of THz spectra has adopted the integrated feature regarding both amplitude and phase such as the combination of absorption rate and refractive index.

Here, a study is proposed to demonstrate that the combination of absorption rate and refractive index can reveal high-dimensional spectral information, which is later abstracted as a feature map and passed to a CNN equipped with efficient channel attention (ECA) mechanism for calibrating the interdependence among data channels [24]. A chemical library containing the solid samples of 20 amino acids is established for this study, and low-lying vibrational modes with respect to the samples are used for the classification of spectra. The proposed method realizes 100% prediction accuracy on validation data, 99.9% and 99.2% on two testing datasets. Additionally, comparisons are made among different classification models and indicate that the proposed method is superior in terms of accuracy and processing speed. These characteristics makes the proposed ECA network an ideal algorithm to be integrated on compact THz sensors.

## 2. Materials and Methods

### 2.1. Experimental Equipment and Sample Preparation

**Figure 1:**
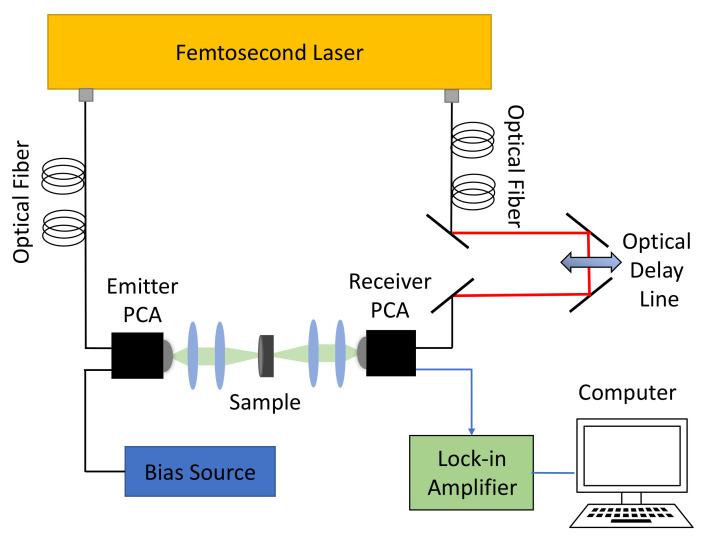
A schematic of the experimental device.

The experimental device includes an ultra-fast femotosecond laser, an optical delay line, emitter and receiver photoconductive antennas (PCAs), a lock-in amplifier, and a computer to control the device and process the signal. The femotosecond laser has a central wavelength of 1560 nm, a pulse width around 100 fs, a repetition rate of 100 MHz, and working power of 80 mW. The laser utilizes doped fiber as gain medium, and the generated power is evenly distributed to two channels, which are used for the generation and sampling of the THz radiation respectively. Single-mode optical fibers are used to transmit laser between modules. An illustration of the experimental device is shown in Figure 1.

As shown in Figure 1, the emitter PCA is connected to a bias source that provides energy for the generation of THz pulses, which have a width of around 1 ps. Afterwards, the emitted THz beam is collimated and focused by two plano-convex lenses, respectively. Then, the beam transmits through the sample and is collimated and focused by two other plano-convex lenses to the receiver PCA, by which the absorption characteristics of the sample are measured. The temporal shape of the received signal is sampled by a optical delay line of 90 ps range and intensified by a lock-in amplifier connected to the computer. The delay line works at 60 Hz, which samples the time–domain THz spectrum 60 times per second. The system achieves a bandwidth of 4.5 THz and a dynamic range of 80 dB.

The 20 amino acid samples used in the experiment were purchased from Shanghai Aladdin Reagent Company. The samples were grounded with a pestle and mortar, and filtered with a 180 mesh sieve to exclude the particles larger than 80 μm so that the scattering effect was attenuated. The samples were then mixed with polyethylene in a 1:1 (*w*/*w*) ratio and pressurized at 30 MPa for approximately 5 min into solid tablets. The tablets were around 1.2 mm thick and 10 mm in diameter. 5 tablets were made for each amino acid, and each tablet was measured by continuous acquisition that lasted 40 s to accumulate about 2400 signals. The measurement chamber was purged with nitrogen gas to reduced the air humidity to 10%, and the experiment was carried out at 17 °C, which was reduced from the room temperature of 25 °C by the nitrogen gas.

### 2.2. Hybrid Spectrum Combined with Absorption Rate and Refractive Index

**Figure 2:**
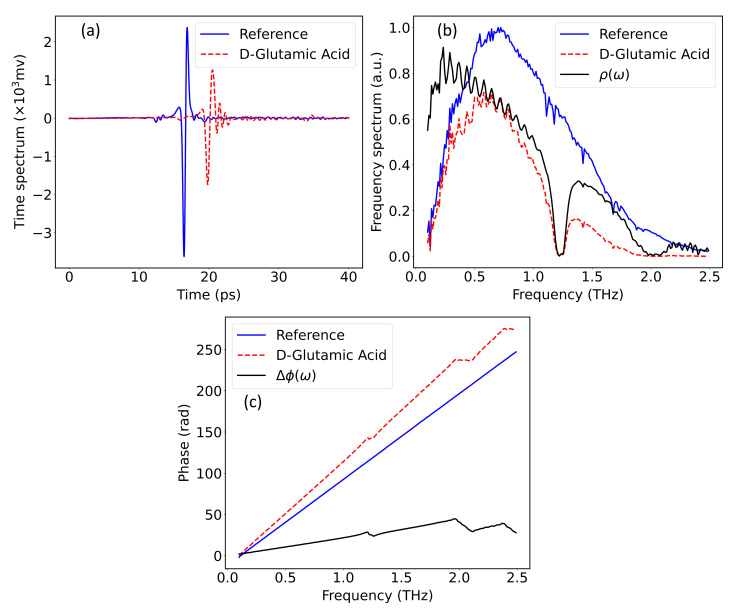
A comparison between the reference signal and the D-Gluatamic acid regarding (**a**) the time-domain spectra, (**b**) the amplitudes of the frequency–domain spectra, (**c**) the phases of the frequency–domain spectra. The solid black curves in (**b**,**c**) denote the amplitude and the phase of the transfer function, respectively.

An advantage of pulsed THz radiation is that the amplitude and the phase of the signal can be extracted simultaneously by Fourier analysis. In particular, the attenuation of amplitude responds to the absorption of the THz wave, and the change in phase reveals the change of refractive index [25]. As shown in Figure 1, this study uses transmission mode to measure the properties of the sample. A reference signal is required to extract the spectral information, explicitly the changes of amplitude and phase, due to the sample. Therefore, the reference signal was measured by removing the sample from the optical path, and the data collected by 10 s continuous acquisition were averaged to reduce the white noise generated by the vibration of the delay line. Meanwhile, the residual noise can be further attenuated with wavelet shrinkage denoising. The wavelet shrinkage denoising follows the idea that wavelet coefficients having small absolute values enclose mostly noise, and the important information is encoded by coefficients having large absolute values. Thereby, removing the small absolute value coefficients and then reconstructing the signal could produce a signal with less noise [26]. Here, a wavelet decomposition with maximum level of 5 is used to denoise the signal, and the sym4 wavelet is selected for the optimal outcome. A comparison between the spectrum of D-Glutamic acid, which was averaged over 40 s continuous acquisition of a single tablet, and the reference signal is shown in Figure 2.

**Figure 3:**
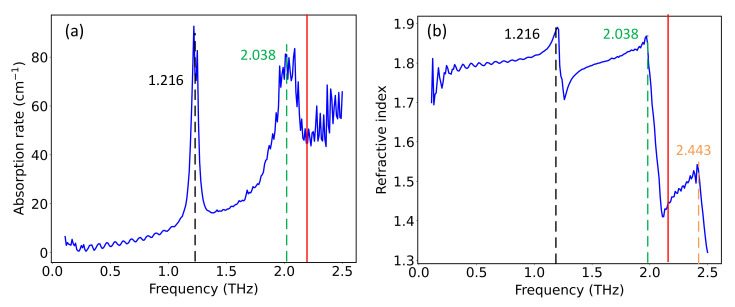
(**a**) The absorption rate of the D-Glutamic acid. (**b**) The refractive index of the D-Glutamic acid. Absorption peaks located at 1.216, 2.038, and 2.443 THz are highlighted by dashed black, green, and orange lines, respectively. The solid red line located at 2.2 THz marks the frequency, after which the absorption rate is no longer in accordance with the actual spectrum. Nevertheless, the refractive index holds with the actual trend after 2.2 THz.

In Figure 2a, the main pulse of the D-Glutamic acid spectrum is delayed by approximately 3 ps from the main pulse of the reference signal, followed by a series of decaying fluctuations. The delay is attributed to the higher refractive index of the sample, and the decaying fluctuations can be explained by the etalon effect by which the THz pulse is reflected by the front and back sides of the sample changeably. The etalon effect also causes baseline fluctuations in the frequency domain, which is shown in Figure 3. The spectral information carried by the sample can be extracted in the frequency domain, as given by Equation (1):

(1) $$F\left\{E\left(t\right)\right\}=\hat{E}\left(\omega \right)=\left|\hat{E}\left(\omega \right)\right|\exp\left(-i\phi \left(\omega \right)\right),$$

where $F\left\{E\left(t\right)\right\}$ denotes the Fourier transform of the time–domain spectrum $E\left(t\right)$, ω denotes the angular frequency, $|\hat{E}\left(\omega \right)|$ and $\phi \left(\omega \right)$ are the amplitude and phase of the frequency–domain spectrum, $\hat{E}\left(\omega \right)$ respectively. The amplitude and phase changes can be obtained from the transfer function, which follows:

(2) $$T\left(\omega \right)=\frac{{\hat{E}}_{samp}\left(\omega \right)}{{\hat{E}}_{ref}\left(\omega \right)}=\rho \left(\omega \right)\exp\left(-i\Delta \phi \left(\omega \right)\right),$$

where $T\left(\omega \right)$ is the transfer function, ${\hat{E}}_{samp}\left(\omega \right)$ is the frequency–domain spectrum of the sample, ${\hat{E}}_{ref}\left(\omega \right)$ is the frequency–domain spectrum of the reference signal, $\rho \left(\omega \right)$ is the amplitude ratio between the sample and reference spectra, $\Delta \phi \left(\omega \right)$ is the phase change caused by propagation through the sample. Figure 2b,c denote the changes in amplitude and phase regarding the D-Glutamic acid spectrum and the reference spectrum. Based on the transfer function and the assumption that the extinction rate is much smaller than the refractive index in the THz band, the absorption rate and refractive index of the sample can be derived from the Fresnel’s law, as given by Equations (3) and (4):

(3) $$n\left(\omega \right)=\frac{\Delta \phi \left(\omega \right)c}{\omega d}+1,$$

(4) $$\alpha \left(\omega \right)=\frac{2}{d}\ln\left\{\frac{4n\left(\omega \right)}{\rho \left(\omega \right){\left[n\left(\omega \right)+1\right]}^{2}}\right\},$$

where $n\left(\omega \right)$ denotes the refractive index, $\alpha \left(\omega \right)$ denotes the absorption rate, *d* is the thickness of the tablet, *c* is the speed of light. Figure 3 gives the absorption rate and the refractive index of D-Glutamic acid sample, where the time-domain spectra in Figure 2 were used in the calculation.

As shown in Figure 3, the absorption peaks of D-Glutamic acid located at 1.216 THz and 2.038 THz are configured by the absorption rate and refractive index, respectively, which can be explained by the Kramers–Kronig relations by which the real and imaginary parts of the complex refractive index are connected [25]. This feature forms the basis of the input block of the neural network in later discussion. It is noticed that the absorption rate in Figure 3a becomes highly compromised after 2.2 THz due to absorption and scattering, and the peak located at 2.443 THz is completely disguised by noise. However, the region after 2.2 THz in Figure 3b is still explicit, and the peak located at 2.443 THz is discernible, as highlighted by the dashed orange line. This reveals that the refractive index can hold spectral features that are not distinguishable from the absorption rate; thus, the combination of the two metrics would give more explicit description of the spectral information. Table 1 gives the absorption peaks of the 20 amino acids referenced from previous research and the measurements in this study. For the rest of the paper, all spectra are cutoff from 0.1 to 2.5 THz, which contains 240 points given the frequency step of 0.01 THz. The spectral information of all the 20 amino acids will be given in Appendix A.

**Figure 4:**
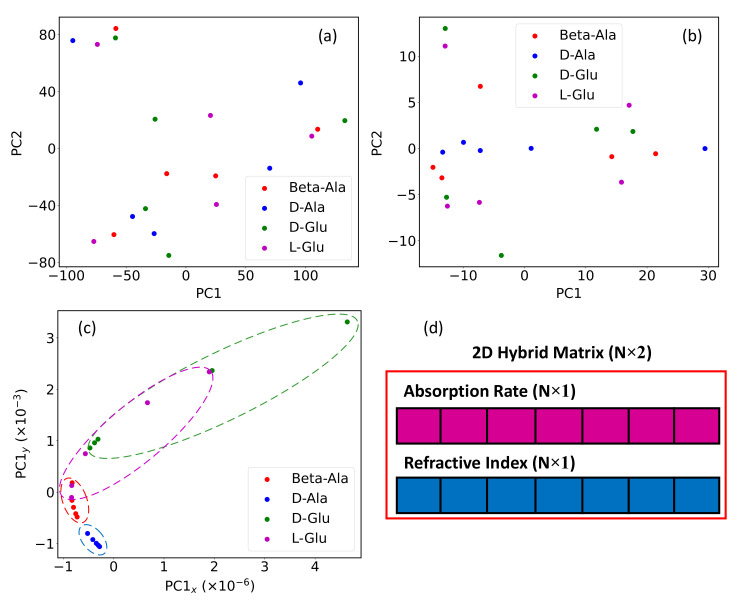
PCA is applied for Beta-Alanine, D-Alanine, D-Glutamic acid, and L-Glutamic acid regarding (**a**) absorption rate, (**b**) refractive index, and (**c**) the 2D hybrid spectrum. Each spot in the graph is generated by averaging the 40 s continuous acquisition of a single tablet. (**d**) illustrates the 2D hybrid spectrum, which is stacked by the absorption rate and refractive index to a N×2 matrix. N is the size of 1D spectrum, which is 240 in this study.

In addition, the effect of combining absorption rate and refractive index can be configured by PCA. As shown in Figure 4a,b, the first two principal components of the absorption rate and the refractive index can not separate different categories. In contrast, after stacking the absorption rate and refractive index to a 2D vector and extracting the first principal component, the points belonging to different amino acids form clusters, as highlighted by dashed elliptic circles in Figure 4c. The hybrid spectrum combined with absorption rate and refractive index is explained by Figure 4d and will be used to classify the amino acids in later discussions.

**Table 1:** The absorption peaks of the 20 amino acids.- represents no specific absorption peak is available in the frequency range.

Amino Acid	Peaks from Literature (THz)	Measured Peaks (THz)
Beta-Alanine	- [27]	-
D-Alanine	2.231 [27]	2.226
L-Alanine	2.231 [27]	2.227
D-Arginine	1.003/1.491 [27]	0.99/1.435
L-Arginine	0.99/1.47 [28]	1.002/1.508
D-Aspartic acid	- [27]	-
L-Aspartic acid	- [27]	-
D-Glutamic acid	1.234/2.031/2.443 [27]	1.216/2.038/2.443
L-Glutamic acid	1.209/2.031 [27]	1.235/1.967
D-Serine	- [27]	-
L-Serine	- [27]	-
DL-Tyrosine	- [27]	-
L-Tyrosine	0.951/1.929/2.083 [27]	0.975/1.929/2.076
Glycine	- [27]	-
L-Leucine	0.85/1.46/1.7/2.17 [29]	0.854/1.48/1.683/2.198
L-Lysine	0.9/2.07 [29]	0.956/2.069
L-Methionine	- [27]	-
L-Threonine	1.42/2.13 [30]	1.418/2.034
L-Tryptophan	1.44/1.851/2.289 [27]	1.447/1.88/2.285
L-Valine	1.7/2.22 [29]	1.678/2.236

### 2.3. Efficient Channel Attention Network

**Figure 5:**
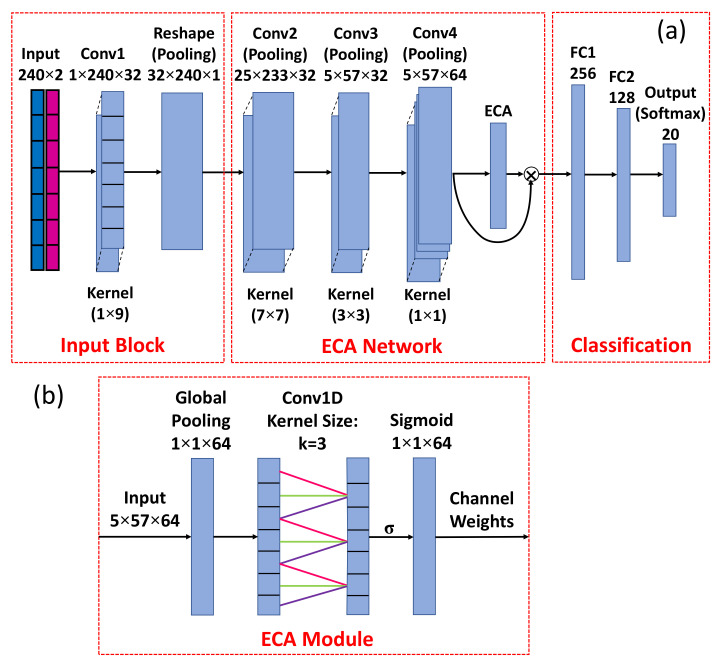
(**a**) A schematic of the ECA network comprised by input block, ECA network, and fully connected layers for classification; (**b**) the structure of the ECA module. The two layers in the middle depict a 1D convolution of the channel-wise data with a kernel size of 3.

In response to classification of the amino acids and inspired by the works in [24,31], a CNN that reshapes the hybrid spectrum to a feature map and identifies it by a convolutional network associated with ECA mechanism is proposed. The structure of the network is illustrated in Figure 5.

As shown in Figure 5a, the hybrid spectrum is passed to a convolutional layer that has 32 filters (Conv1), and the outputs of the layer are 32 filtered versions of the input signal. These signals are then stacked to a 2D matrix, so that the input to the next layer Conv2 is a single feature map instead of 32 distinct filtered signals. The input and reshaping layers are combined as the input block. Layers Conv2 and Conv3 use 2D filters to capture relationships across the filtered signals produced by Conv1, and in turn output 32 channels, respectively. Layer Conv4 uses 1×1 kernel to increase the number of channels to 64, which extracts more channel-wise information for ECA module. As shown in Figure 5b, the ECA module is composed of a global pooling layer to reduce the dimension to 1×1×64, a 1D convolutional layer to implement the cross-channel interaction, and an activation layer using sigmoid function to render nonlinearity to channel weights (attention coefficients, coefficients to enforce the interconnection between neighboring channels). A channel-wise multiplication is performed to the output of Conv4 and the attention coefficients, so that the interdependencies between channels are substantially handled. Two fully connected layers of sizes 256 and 128 are used to reduce the number of hyper parameters passed from ECA network, followed by a dense layer using softmax activation function, which outputs the probability of each category, for classification. Batch normalization is applied after each convolutional layer to standardize the outputs of the layer for each mini batch. Pooling layer is added afterwards to reduce the output dimension.

The detailed description of ECA can be found in [24]. A brief introduction regarding the attention mechanism and the adaptive kernel size is given here. To address the interaction between channels in a CNN, a mechanism to recalibrate the weights of different channels is required. As mentioned by [24], ECA is an efficient method to calculate the channel weights without dimensionality reduction. The estimation of the weights follows Equation (5):

(5) $$w_{i}=\sigma \left(\sum_{j=1}^{k}w^{j}y_{i}^{j}\right),y_{i}^{j}\in \Omega_{i}^{k},$$

where $y_{i}^{j}$ is the input from the adjacent channel *j* of channel *i*, $w^{j}$ is the weight for $y_{i}^{j}$, *k* is the number of adjacent channels, and $\Omega_{i}^{k}$ is the set of neighboring channels. This mechanism not only handles the cross-channel interactions but also avoids complete independence among different groups. As aforementioned, attention are calculated by a 1D convolution with a kernel size of *k*. Depending on the total number of channels, *C*, *k* can be adaptively adjusted for the optimal performance. Equation (6) gives the relationship between *C* and *k*:

(6) $$k={\left|\frac{\log_{2}\left(C\right)}{\gamma }+\frac{b}{\gamma }\right|}_{odd},$$

where γ and *b* are fitting factors, which are set as γ=2, b=1 in this study; ${\left|\;\cdot \;\right|}_{odd}$ indicates the nearest odd number. According to Equation (6), *k* is equal to 3 for the 64 channels given by Conv4.

### 2.4. Training Details

To make the training data, 100 signals of a tablet are averaged for a single record. Thereby, there are about 24 records for each tablet and 120 records for each amino acid. For the testing data, the signals are averaged 20 and 10 times to form two datasets (Average20 and Average10) that are noisier than the training data, which are designed to test the robustness of the model. The training data were shuffled, and 20% of the data were assigned for validation. The training used stochastic gradient decent (SGD) with an initial learning rate of $10^{-3}$, a learning rate decay of $10^{-5}$, a momentum of 0.9, Nesterov accelerated gradient for faster convergence [32], and a batch size of 128. Cross-entropy loss function was used to measure the distance between the predicted and target labels during the training process. The training ran for 300 epochs with the initial learning rate, and would come to an early stop if the loss did not decrease for 30 consecutive epochs. Then, the training ran for other 100 epochs with a learning rate of $10^{-4}$ to fine-tune the model. All programs ran on a PC equipped with a RTX 2070 GPU and an 8-core Intel(R) i7 CPU.

## 3. Results

### 3.1. Metrics

The accuracy (*Acc*) was used to evaluate the classification performance of the model, and precision (*Pr*) is used to evaluate the classification performance for each category, as shown in Equations (7) and (8):

(7) $$Acc=\frac{\sum_{i}n_{ii}}{\sum_{i,j}n_{ij}},$$

(8) $$Pr_{i}=\frac{n_{ii}}{\sum_{j}n_{ij}},$$

where *i* refers to the index of the label, *j* denotes the index of the predicted label. $n_{ij}$ represents the number of items belong to category *i* predicted as category *j*. Similarly, $n_{ii}$ represents the number of correct predictions associated with category *i*.

Here, the number of hyper parameters (#.Param.), the floating point operations per second (FLOPs), training time, and processing speed (test rate, frame per second, fps) are used to evaluate the efficiency of the model.

### 3.2. The Effects of Hybrid Spectrum and ECA Module

**Figure 6:**
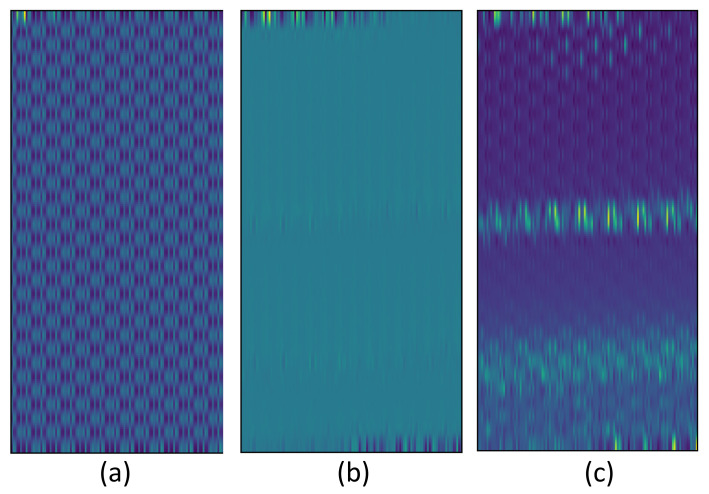
The feature maps of D-Glutamic acid generated with (**a**) absorption rate, (**b**) refractive index, and (**c**) 2D hybrid spectrum.

To demonstrate the effectiveness of the proposed network, the effect of hybrid spectrum and ECA module are studied explicitly. First, the absorption rate and the refractive index were separately taken as the training data, and the model was trained as aforementioned. The feature maps produced by the input block of the trained models are shown in Figure 6, where the vertical axis of the image corresponds to the data size of each channel and the horizontal axis of the image corresponds to the number of channels.

As seen in Figure 6a,b, the feature maps generated with only absorption rate or refractive index do not have obvious patterns cross different channels. In contrast, the feature map generated with the hybrid spectrum in Figure 6c encloses patterns that signify the connection among channels, so that the following convolutional layers could sufficiently extract the high-dimensional features by applying multiple channels and make more accurate predictions. The results regarding different inputs are given in Table 2, where the accuracy of ECA network with hybrid spectrum as input is 1.2% and 3.4% higher than those loaded with only absorption rate and refractive index on Average20 dataset, and 1.9% and 5.6% higher on the Average10 dataset.

**Figure 7:**
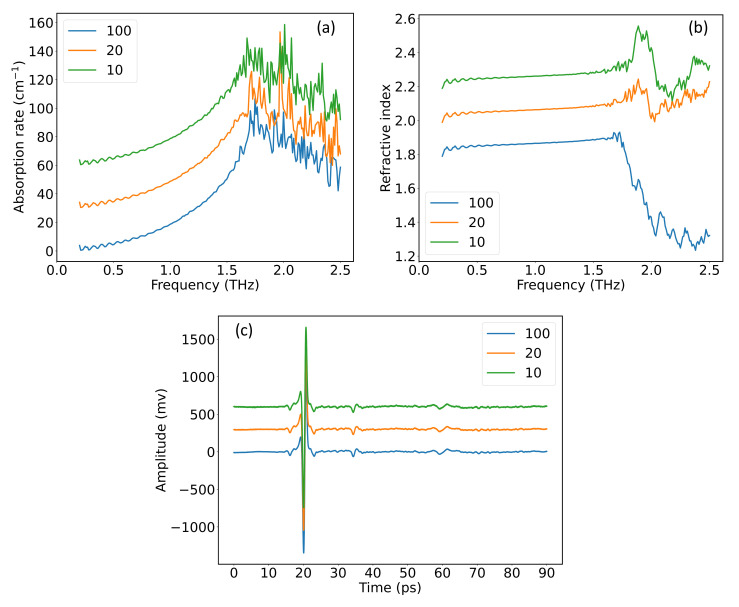
(**a**) Absorption rate, (**b**) refractive index, and (**c**) time-domain spectrum of D-Serine in training dataset (100) and testing datasets (20 and 10). Offset on the *y*-axis was used for clarity.

The effect of ECA module is tested by ablation study, where the Conv4 layer and ECA module are removed to test the feasibility of the remaining network, which is denoted as plain CNN in Table 2. The classification accuracy of ECA network is 0.2% higher than that of plain CNN on the Average20 dataset, and 0.3% higher on the Average10 dataset. The classification precision of most categories is also higher for the ECA network. However, the precision of D-Serine is the lowest among all categories, especially for classification by absorption rate or refractive index. This can be explained by Figure 7.

As shown in Figure 7a, the higher bound of high-SNR region is around 1.7 THz, followed by the low-SNR features that vary largely for different average times. In Figure 7b, the slope of refractive index is more evident in the training data than in the testing data. This brings difficulties to the trained model to identify the features in testing data, and thus lowers the classification precision. To conclude, the ECA network with hybrid spectrum as input can sufficiently improve the results of classification and achieve considerable accuracy in complex scenarios such as D-Serine.

### 3.3. Compare with Other ECA-Based Networks

ECA-based networks referred from [24,33] are compared with the ECA network. The network in Figure 5a first substituted the ECA network for the aforementioned networks, then trained them with the hybrid spectrum. As seen from Table 2, the accuracy of our ECA network is 11.3% higher than the ECA-DDCNN proposed by [33], 6.8% and 7.7% higher than the ECA-Resnet50 and ECA-Resnet101 proposed by [24] for the Average20 dataset. For the Average10 dataset, the accuracy is 26.2% higher than ECA-DDCNN, 10.5% higher than ECA-Resnet50, and 15.7% higher than ECA-Resnet101. A comparison among different networks is given in Table 3.

**Figure 8:**
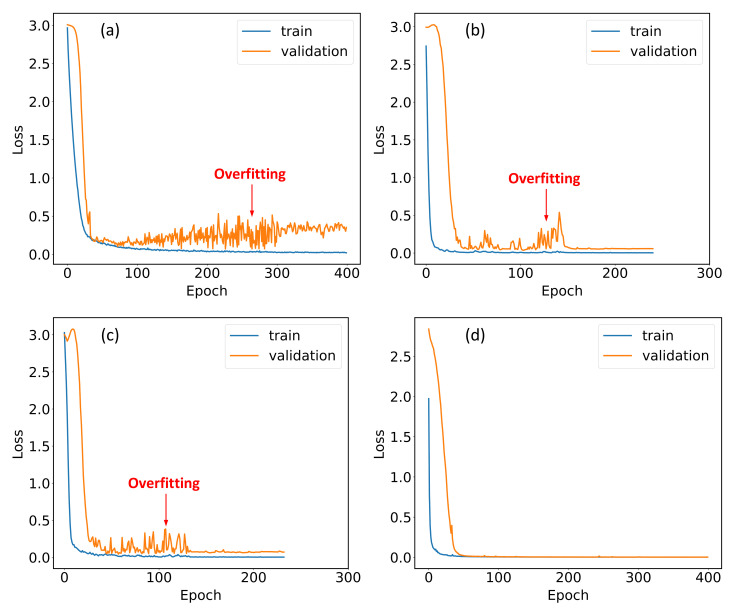
Training histories of (**a**) ECA-DCNN, (**b**) ECA-Resnet50, (**c**) ECA-Resnet101, and (**d**) ECA network.

As illustrated by Table 3, the depths and number of parameters of previously proposed ECA-based networks are much greater than the ECA network, which makes the optimization more complicated, as verified by the longer training times. As the training dataset contains about 2400 records of data, the deeper models may experience overfitting in which the model is too closely aligned to a limited set of data points during the training and hence is less adaptive to the data outside training dataset. Figure 8 displays the training history of each ECA-based network, where the divergence between losses belong to training data and validation data indicates the occurrence of overfitting.

As shown by Figure 8, all the deep networks experienced overfitting during the training, which from one aspect explains their lower classification accuracy.

### 3.4. Compare with Other Methods for Amino-Acid Classification

In previous studies of amino-acid classification, Ref. [23] proposed a network combining CNN and bidirectional gated recurrent network (BiGRU) to trace the dynamic changes of THz time spectrum, and [22] proposed a CNN inspired by LeNet5 to classify the 2D images composed by frequency spectra [34]. As shown in Table 2, the accuracy of the CNN is 12.5% lower than ECA network on Average20, and 23% lower on Average10. Meanwhile, the accuracy of CNN-BiGRU is 0.2% lower than the ECA network on Average20, but 0.1% higher than ECA network on Average10. However, this can be explained by Figure 7, where time spectrum only changes by noise level but maintains all the dynamic features for different average times; on the contrary, the frequency spectrum differs largely for different average times. This helps CNN-BiGRU achieve higher precision for D-Serine. In fact, the classification accuracy of ECA network on 19 amino acids excluded Serine is 99.95% for Average20 and 99.68 for Average10. In contrast, the accuracy of CNN-BiGRU is 99.75% for Average20 and 99.45% for Average10. In real-world applications, the samples may be contained in packages, and thus the time spectrum varies due to shapes and materials of the packages. Meanwhile, the frequency spectrum is less influenced by the environments and hence is widely used in non-destructive inspection [35]. In this regard, the ECA network is more practical in the real world due to its robustness on noisy test data and higher processing speed, i.e., the test rate of ECA network is 3782.46 fps, compared to 84 fps of CNN-BiGRU.

## 4. Discussion

In this paper, the combination of absorption rate and refractive index for extracting high-dimensional information from the THz spectrum was proposed, and an ECA-based CNN was designed to classify the combined spectrum with high accuracy. Compared with other ECA-based networks, the proposed ECA network avoided overfitting during the training and achieved higher accuracy. The accuracy of the proposed method is higher than a previous method that classifies solely absorption spectrum [22], and slightly lower than the method working with time spectrum due to the changed features among frequency–domain data [23]. The proposed method achieved the highest processing speed among all the methods and smaller size than other ECA-based networks, which makes it a viable option for integration to embedded systems. However, this paper only discussed the classification of pure chemicals, but in real world samples are usually compounds, therefore extending the ECA network to compound classification will be the next step of our investigation.

## Figures and Tables

**Table 2:** The precision and accuracy of classification of 20 amino acids on Average20 and Average10 datasets. From a to h are: a. ECA network with absorption rate as input; b. ECA network with refractive index as input; c. plain CNN with hybrid spectrum as input; d. ECA-DDCNN [33] with hybrid spectrum as input; e. ECA-Resnet50 [24] with hybrid spectrum as input; f. ECA-Resnet101 [24] with hybrid spectrum as input; g. CNN-BiGRU referred from [23]; h. CNN referred from [22]. Ours denotes the ECA network with hybrid spectrum as input. The values in the table are percentages.

20	Beta-Ala	D-Ala	L-Ala	D-Arg	L-Arg	D-Asp	L-Asp	D-Glu	L-Glu	D-Ser	L-Ser	DL-Tyr	L-Tyr	Gly	L-Leu	L-Tys	L-Met	L-Thr	L-Try	L-Val	Acc
a	99.2	100	99.8	99.6	100	99.6	100	100	99.8	80.8	97.8	99.4	100	99.8	100	99.8	100	99.6	100	99.8	98.7
b	100	99.6	100	90.6	99.3	100	99.6	100	99.8	54.9	95.4	100	99.8	100	100	99.8	91.3	100	99.8	99.1	96.5
c	99.8	100	99.8	100	99.8	99.8	100	100	99.8	98.0	98.3	99.1	99.6	100	100	99.8	100	100	100	99.8	99.7
d	98.9	98.7	99.8	84.1	100	51.9	49.4	100	97.8	99.6	98.1	97.6	100	100	98.5	99.6	99.8	98.1	100	100	88.6
e	99.6	96.5	89.9	99.1	99.4	91.7	97.2	99.8	99.8	99.4	99.6	98.9	98.3	98.7	100	99.8	98.9	98.2	97.4	98.5	93.1
f	99.6	94.3	92.3	99.3	99.1	93.2	79.7	99.6	99.6	99.8	98.9	98.7	97.8	99.6	100	99.8	98.5	98.2	97.4	98.7	92.2
g	75.9	90.2	83.3	95.9	94.5	87.5	84.3	98.2	97.6	53.2	79.2	91.9	96.9	82.0	94.7	89.9	90.0	96.9	93.7	91.0	87.4
h	99.8	99.8	99.4	100	100	98.5	99.8	100	100	99.3	99.3	99.1	100	99.1	100	100	100	100	99.6	99.8	99.7
a	98.5	99.4	98.0	99.3	99.5	99.1	99.2	99.4	99.4	65.7	96.3	98.7	99.8	98.9	99.9	99.2	100	98.9	98.0	98.9	97.3
b	100	98.6	98.3	77.1	94.5	99.9	98.1	100	99.6	57.1	95.5	99.2	99.6	100	100	100	77.2	99.9	96.1	97.3	93.6
c	99.6	99.6	99.0	99.4	99.2	99.6	99.7	100	99.4	92.2	96.5	97.8	99.4	99.8	99.6	99.9	100	100	98.4	98.4	98.9
d	88.3	97.3	97.2	63.7	99.2	54.8	67.0	100	61.5	88.7	52.0	64.2	99.9	99.4	80.3	96.4	98.8	67.2	99.1	99.5	73.0
e	96.0	93.0	74.4	93.9	99.2	78.5	74.5	99.2	98.8	98.9	97.8	92.2	96.8	96.0	99.2	98.3	94.9	97.9	95.8	97.1	88.7
f	90.6	88.2	82.8	97.2	98.5	61.9	34.7	98.6	97.5	95.7	60.5	90.6	96.0	97.8	98.5	96.3	93.9	95.1	95.4	96.2	83.5
g	59.7	79.5	71.3	87.6	88.6	78.8	68.9	89.9	88.5	48.8	61.3	79.3	92.7	68.2	81.4	67.9	82.9	90.4	83.9	83.7	76.2
h	99.8	99.3	98.1	100	100	97.9	99.6	100	100	98.1	99.0	98.3	100	98.0	100	100	100	100	99.3	99.2	99.3
**ours**	**99.5**	**100**	**98.6**	**100**	**99.4**	**99.7**	**99.7**	**100**	**99.6**	**90.3**	**99.1**	**99.5**	**99.8**	**99.7**	**99.9**	**100**	**100**	**99.8**	**99.6**	**99.7**	**99.2**

**Table 3:** Comparison of different models in terms of network depth, network parameters (#.Params), floating point operations per second (FLOPs), training time, and processing speed on test data (test rate).

	Depth	#.Params	FLOPs	Training Time	Test Rate
ECA-DCNN [33]	125	7.33 M	421.42 M	21.60 min	943.93 fps
ECA-Resnet50 [24]	67	24.14 M	588.23 M	11.56 min	1134.35 fps
ECA-Resnet101 [24]	134	43.21 M	675.45 M	19.74 min	675.45 fps
**ECA network (ours)**	**4**	**4.72 M**	**64.61 M**	**3.67 min**	**3782.46 fps**
CNN-BiGRU [23]	-	3.23 M	6.31 M	169.63 min	84.00 fps
CNN [22]	-	0.93 M	103.07 M	11.4 min	1887.13 fps

**Figure A1:**
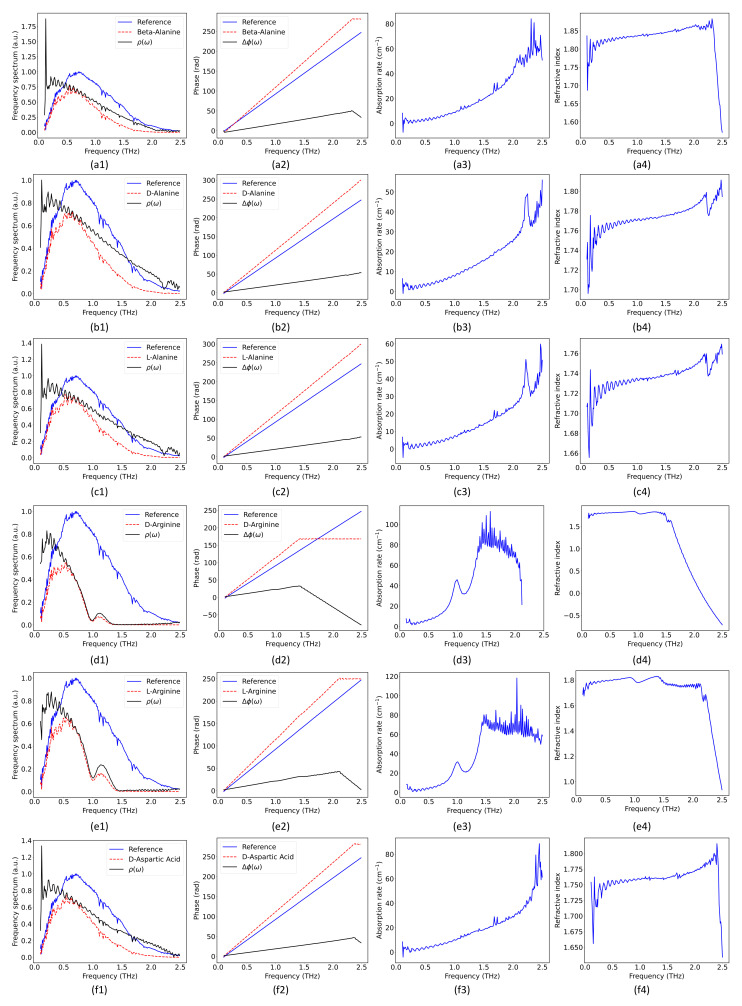

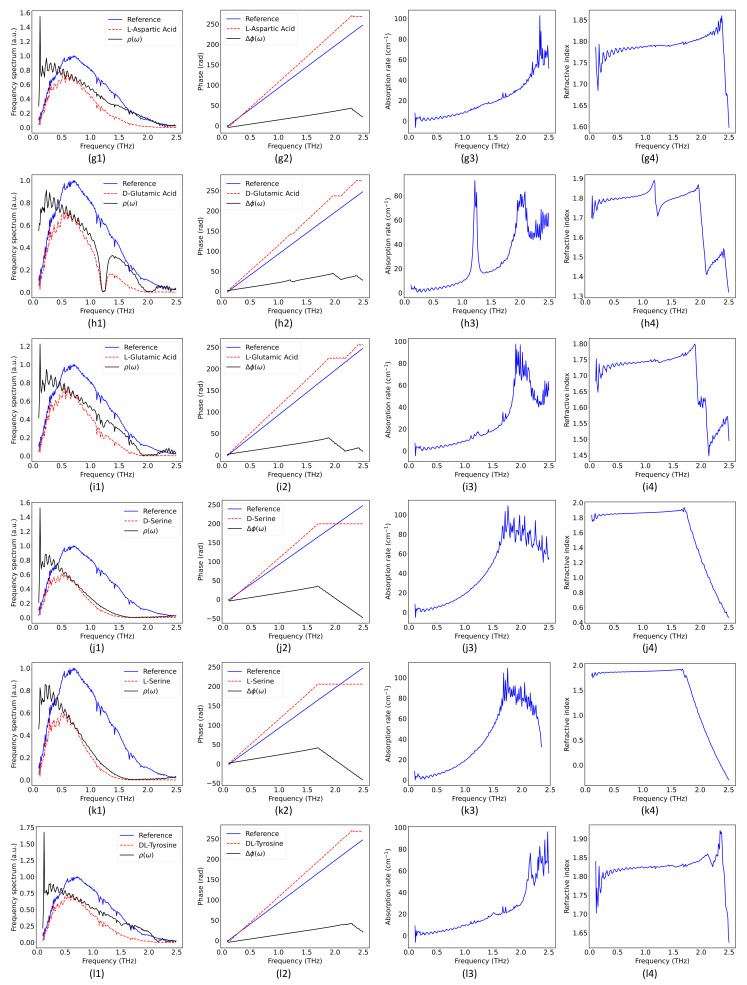

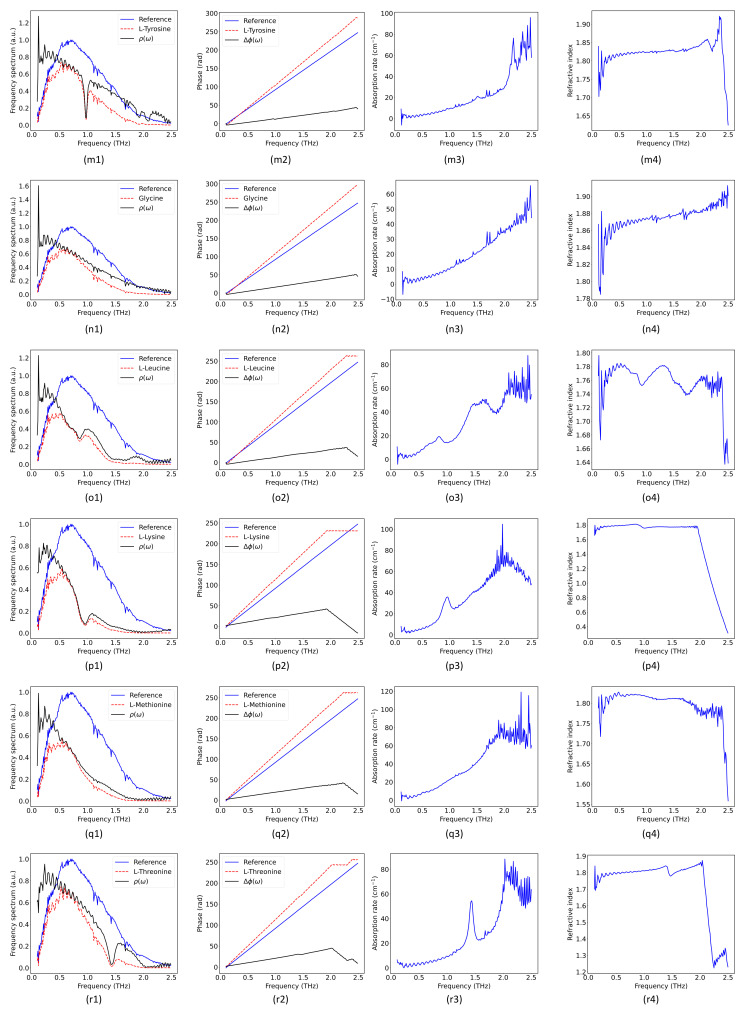

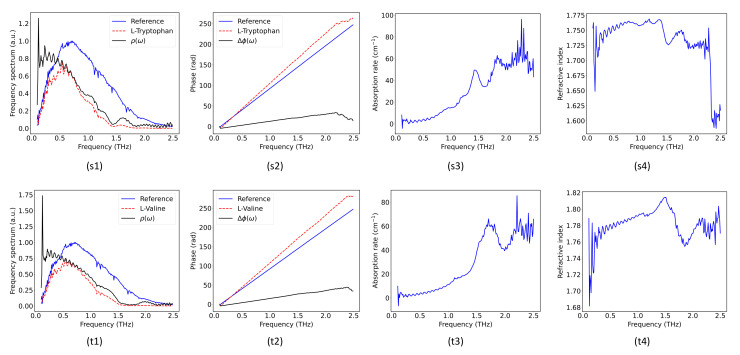
(**a**–**t**) are Beta-Alanine, D-Alanine, L-Alanine, D-Arginine, L-Arginine, D-Aspatic acid, L-Aspatic acid, D-Glutamic acid, L-Glutamic acid, D-Serine, L-Serine, DL-Tyrosine, L-Tyrosine, Glycine, L-Leucine, L-Lysine, L-Methionine, L-Threonine, L-Tryptophan, and L-Valine, respectively. In each group, 1 and 2 are the amplitude and phase of the frequency–domain spectrum; 3 and 4 are the absorption rate and the refractive index of the sample.

## Data Availability

Data underlying the results presented in this paper are not publicly available at this time but may be obtained from the authors upon reasonable request.

## Appendix A

To get an intuitive picture of the classification problem discussed in main text, the spectral information described by Figure 2b,c as well as Figure 3a,b is given regarding the 20 amino acids used in the classification, as shown in Figure A1.
